# A channelopathy mutation in the voltage-sensor discloses contributions of a conserved phenylalanine to gating properties of Kv1.1 channels and ataxia

**DOI:** 10.1038/s41598-017-03041-z

**Published:** 2017-07-04

**Authors:** Sonia Hasan, Cecilia Bove, Gabriella Silvestri, Elide Mantuano, Anna Modoni, Liana Veneziano, Lara Macchioni, Therese Hunter, Gary Hunter, Mauro Pessia, Maria Cristina D’Adamo

**Affiliations:** 1 Department of Physiology, Faculty of Medicine, Kuwait University, Safat, 13110 Kuwait; 2 Section of Physiology and Biochemistry, Department of Experimental Medicine, School of Medicine, University of Perugia, Perugia, Italy; 3 Institute of Neurology, Catholic University of Sacred Heart, Fondazione Gemelli, Rome, Italy; 4 Institute of Translational Pharmacology, National Research Council of Italy, Rome, Italy; 5 Faculty of Medicine & Surgery, Department of Physiology & Biochemistry, University of Malta, MSD 2080 Msida, Malta

## Abstract

Channelopathy mutations prove informative on disease causing mechanisms and channel gating dynamics. We have identified a novel heterozygous mutation in the *KCNA1* gene of a young proband displaying typical signs and symptoms of Episodic Ataxia type 1 (EA1). This mutation is in the S4 helix of the voltage-sensing domain and results in the substitution of the highly conserved phenylalanine 303 by valine (p.F303V). The contributions of F303 towards K^+^ channel voltage gating are unclear and here have been assessed biophysically and by performing structural analysis using rat Kv1.2 coordinates. We observed significant positive shifts of voltage-dependence, changes in the activation, deactivation and slow inactivation kinetics, reduced window currents, and decreased current amplitudes of both Kv1.1 and Kv1.1/1.2 channels. Structural analysis revealed altered interactions between F303V and L339 and I335 of the S5 helix of a neighboring subunit. The substitution of an aromatic phenylalanine with an aliphatic valine within the voltage-sensor destabilizes the open state of the channel. Thus, F303 fine-tunes the Kv1.1 gating properties and contributes to the interactions between the S4 segment and neighboring alpha helices. The resulting channel’s *loss of function* validates the clinical relevance of the mutation for EA1 pathogenesis.

## Introduction

Voltage-gated potassium channels (Kv) play key roles in neurotransmission and nerve cell physiology^1^ and are of high therapeutic relevance^2^. Strategically clustered at critical subdomains such as the axon initial segment^3^, juxtaparanodal regions of the Ranvier’s node^4^, as well as in synaptic nerve terminals^5^, Kv1 channels are known key regulators of excitability. They open at voltages close to action potential (AP) threshold to reduce excitability by limiting nodal re-excitation^6^, shaping the presynaptic AP waveform and controlling transmitter release at synaptic terminals^7^. Accordingly, mutations that result in dysfunctional Kv1.1 channels are likely to result in impairments in neuronal excitability and neurotransmission. Of particular clinical importance is the Kv1.1 channelopathy that is predominantly linked to Episodic Ataxia type 1 (EA1). EA1 is an autosomal dominant disorder characterized by frequent, albeit brief, attacks of uncoordinated movements (ataxia) and involuntary repetitive muscular contractions (myokymia). The disorder presents primarily by means of heterozygous point mutations in the *KCNA1* (Kv1.1) gene, located on chromosome 12p13^8–10^. Kv1.1 co-assembles with α-subunits of other members of the Kv1 family to form heterotetrameric channels with biophysical and pharmacological properties that are distinct from homotetramers made up of their contributing subunits^11–16^. Both in the periphery^17, 18^ and in the brain^19, 20^ Kv1.1 is mostly found co-assembled with Kv1.2 subunits. A mutation in Kv1.1 affects the function of the Kv1.1/1.2 heteromeric channel to which they contribute^21^. A number of functional studies on Kv1.1 mutations all report *loss of function* of the channel^1, 8, 21–29^. Yet, despite this consensus, phenotypic heterogeneity such as severity of ataxia and presence of additional features such as epilepsy and hyperthermia exist^24, 30, 31^ and may be connected to differences in mechanisms of dysfunction, altered specific channel parameters or epigenetics^30, 32–34^.

**Figure 1:**
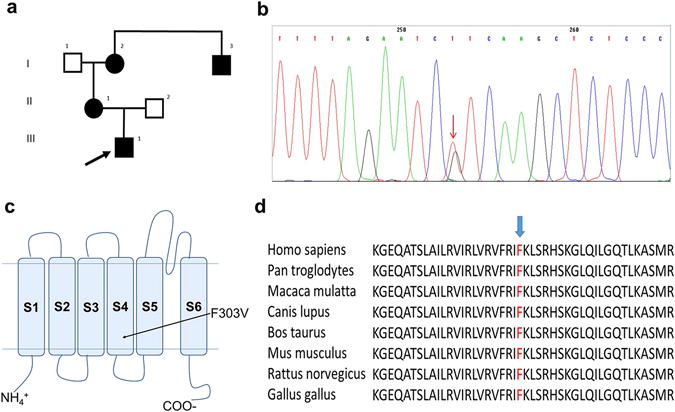
Detecting and analyzing the mutation. (**a**) Pedigree of patient’s family. The arrow indicates the proband, black symbols indicate affected subjects. (**b**) DNA sequencing chromatogram of a section of *KCNA1* (Kv1.1) exon 2 showing the heterozygous mutation segregating in the family. The arrow indicates the c907 T > G nucleotide substitution. (**c**) Schematic topology model of the human Kv1.1 subunit showing approximate site for the F303V mutation in the S4 segment. (**d**) Alignment of the region of interest for Kv1.1 channels from various species using MUSCLE 3.6. The arrow indicates the affected phenylalanine.

Structurally, Kv channels are composed of four subunits each of which contains six transmembrane-spanning segments (S1 through S6) with cytoplasmic N- and C-terminal domains (Fig. 1c). The S1–S4 segments comprise the voltage-sensing domain, which senses the membrane potential and controls the gating of the pore domain (S5–S6). Kv1.1 α-subunits make up channels that mediate the non-inactivating outward delayed-rectifier potassium current^14^. The positively charged amino acids in the S4 segment play a central role in the voltage-dependence of Kv1.1 channels. In particular, the characteristic low activation threshold and distinct activation-deactivation kinetics features^12, 14^ of the channel are crucial for an immediate outward current response to fast depolarizing stimuli, allowing for an effective prevention of excessive neuronal excitation^16^. It has been proposed that the activated state (*i.e*., the upstage) of the voltage-sensors is stabilized by interactions between a highly conserved phenylalanine residue located in segment S2 (F233) and its hydrophobic neighbors, and that mutations of these hydrophobic residues specifically destabilize the open-state and cause a dramatic acceleration in deactivation gating kinetics^35^. Although several studies have focused on the role of F233 to *Shaker*-like channel gating^36–39^, the contribution of the highly conserved phenylalanine residues located within segment S4 is not fully understood. Here, we identified a new mutation in the Kv1.1 channel of family members displaying typical symptoms of EA1 that changes the highly conserved F303, located adjacent to the positively charged residue (K304) in the S4 segment, into a valine (Fig. 1b-d). What functional role does this F303 residue play? The assessments of the biophysical properties of the channel in which F303 was replaced by a valine indicated that the voltage-dependence and gating kinetics of both homomeric and heteromeric Kv1.1 and Kv1.1/1.2 channels are remarkably affected, providing novel insights into both the role of F303 in channel gating and the mechanisms accounting for the neuronal hyperexcitability phenotype displayed by the patient.

## Methods

### Clinical and neurophysiologic examination

Studies involving the proband and his family complied with the Helsinki Declaration and was approved by the Ethical Committee of Catholic University of Sacred Heart, Fondazione Gemelli, Rome, Italy. Written informed consent for participation and publishing were obtained from all subjects (and both parents on behalf of the proband aged <18 years) included in the study.

The neurophysiologic evaluation included sensory and motor nerve conduction studies combined with the measurement of F-wave latencies. Patients were examined in a warm comfortable room; the skin temperature was maintained above 32 °C. Motor nerve conduction studies were carried out on ulnar and tibial nerves. The stimulus was an electrical square pulse of 0.2 ms; current intensity was 150% of that needed to obtain maximal Compound Muscle Action Potential (CMAP) amplitude. The position of the recording electrodes was considered optimal when the CMAP showed maximal amplitude and negative onset. As for ulnar nerves, the recording monopolar surface electrode was placed on *abductor digiti quinti* and referred to the first phalanx of the fifth finger. The CMAP of the tibial nerves was recorded from *abductor hallucis* and referred to the metatarsal-phalangeal joint of the big toe. Sensory nerve conduction studies were carried out on the sural nerve. It was stimulated antidromically: the recording surface electrode was placed under the lateral malleolus bone and the stimulating electrode was kept at a distance of 12 cm from the active recording electrode.

### Genetic analysis

DNA was isolated from peripheral blood using standard methods. *KCNA1* coding regions and exon-intron boundaries were amplified by polymerase chain reaction (PCR) using the following primer pairs:

EA1-F21: AGAAGCAGAGAGGGTGGCAG

EA1-REV2: AAGAAGTCCGTCTTGCTGGG

and

EA1-F1/7B: CCACGGTCATCTACAATTCCAA

EA1-REV: CTTGAAAGCTTCTGGTTCACCACC

To detect the genetic defect underlying the pathology, the PCR products were sequenced using an automated Sanger dideoxy method. The obtained DNA fragments were sequenced by Eurofins MWG Operon Sequencing Service (http://www.mwg-biotech.com/). *In silico* prediction tools PolyPhen (http://genetics.bwh.harvard.edu/pph/) and SIFT (http://sift.jcvi.org/) were used to predict the pathogenicity of the variants found.

### Production of *KCNA1* constructs

Human Kv1.1 cDNA was subcloned into a pBF oocyte expression vector. The mutation p.F303V was introduced by site-directed mutagenesis performed according to the QuikChange protocol (Stratagene, La Jolla, CA) and was verified by automated sequencing. The concentration of the *in vitro* transcribed cRNA was quantified by electrophoresis with ethidium bromide staining and spectrophotometric analysis.

### Heterologous expression of *KCNA1*

Wild-type and mutant channels were expressed in *Xenopus laevis* oocytes as described in D’Adamo *et al*., 1998^23^. All animal handling was in accordance with international standards of animal care, the Italian Animal Welfare Act, approved by the local Veterinary Service Authority, and the NIH Guide for the Care and Use of Laboratory Animals. *Xenopus laevis* were deeply anesthetized with an aerated solution containing 3-aminobenzoic acid ethyl ester methanesulfonate salt (5 mM) and sodium bicarbonate (60 mM), pH 7.3. Stage V–VI *Xenopus Laevis* oocytes were isolated, digested with collagenase, each injected with 50 nl cRNA and incubated at 16 °C in ND96 solution containing (mM): NaCl 96, KCl 2, MgCl_2_ 1, CaCl_2_ 1.8, HEPES 5, gentamicin 50 μg/ml. *Xenopus laevis* underwent no more than two surgeries, separated by at least 3 weeks.

### Electrophysiology

Two-electrode voltage-clamp recordings (TEVC) were performed as previously described^23^. Briefly, whole-cell currents were recorded using TEVC on oocytes at ~22 °C. Recordings were made on days 1 to 7 after cRNA injection, and using a GeneClamp 500 amplifier (Axon Instruments, Foster City, CA) interfaced to a PC computer with an ITC-16 interface (InstruTech, Port Washington, NY). Microelectrodes were pulled to a tip resistance of <1 MΩ and backfilled with 3 M KCl. The extracellular solution contained (mM): NaCl 96, KCl 2, MgCl_2_ 1, CaCl_2_ 1.8, HEPES 5, pH 7.4. Recordings were filtered at 2 kHz and acquired at 5 kHz with Pulse software and analyzed with either PulseFit (HEKA, Germany) or Origin 8 (OriginLab, Northampton, MA). Leak and capacitative currents were subtracted using a P/4 protocol.

### Structural analysis

The coordinates for rat Kv1.2 channel were taken from PDB entry 3LUT^40^. To locate the position of the mutated residue in the rat Kv1.2 channel structure, sequence alignments were performed with ClustalW using the following channel homologs: human Kv1.1 (3736), *Shaker* (P08510), and Kv1.2/2.1 Chimera (P62483). The final sequence alignment was further refined using Muscle 2.6^40^. EA1 mutations were performed *in silico* by substituting the wild type residue with the desired mutation. The human Kv1.1 structural homology model was constructed utilizing the Swiss-Model server^41–43^ and amino acid sequence NP_000208 with the Kv1.2 structure, 3LUT, serving as template (above 84% sequence homology). Amino acid interaction distances were measured using PyMOL^44^.

### Statistical Analysis

Statistical analysis was performed using the software programs Prism 6 (GraphPad Software, San Diego, CA) and Origin 8 (OriginLab, Northampton, MA). All data are shown as mean ± standard error (SE). Observed differences were evaluated by two-tailed unpaired Student’s t-test and were considered significant if p < 0.05.

### Data Availability

The datasets generated during and/or analysed during the current study are available from the corresponding author on reasonable request.

## Results

### Clinical Description

All the symptomatic family members referred since childhood the occurrence of brief episodes, lasting a few minutes or even less, characterized by dizziness, gait unsteadiness and speech impairment modifications. These episodes, usually triggered by physical exercise or emotional stress, were mild and infrequent in the oldest patients (pt I2 and I3 in Fig. 1a), while occurring more frequently in pt II1 and in her 14-year-old son III1 (Fig. 1a), the family proband, in which both the intensity and the recurrence of ataxic episodes were disabling.

Interictal neurological examination showed a fine postural tremor distally in the upper extremities both in the proband and his mother pt II1, showed nystagmus in the extreme lateral gaze and postural tremor in the upper limbs. Finally pt I2, his maternal grandmother, who is the oldest affected family member, manifested rare facial myokymia, diffuse postural limb tremor and some difficulties in tandem walking. All patients had normal muscle tone and trophism, normal reflexes and no sensory deficit. In the proband, we were able to trigger a brief episode of gait ataxia and dysarthria, lasting a few seconds that was elicited by physical exercise (short run).

**Figure 2:**
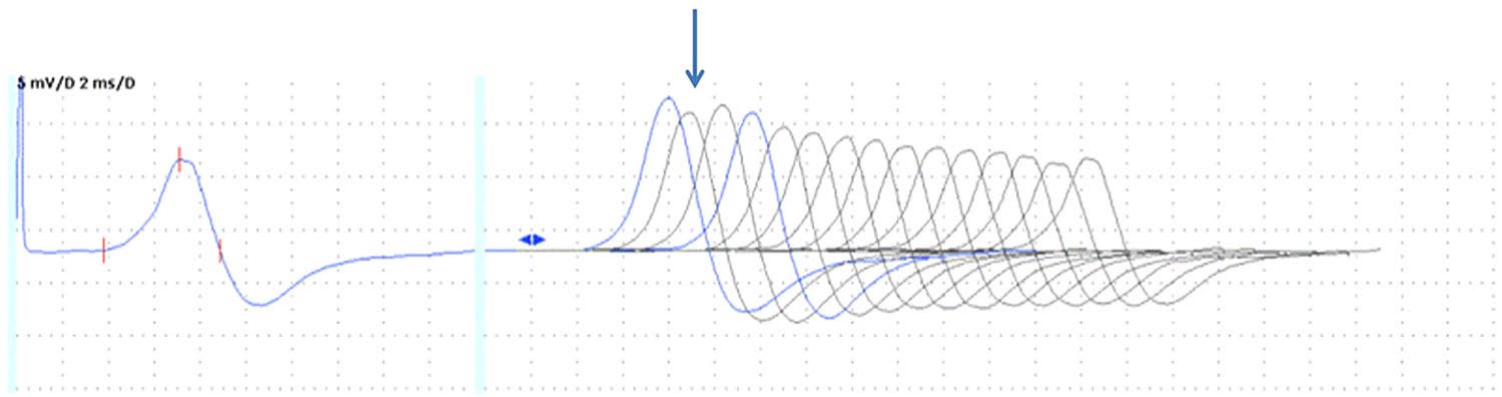
CMAPs evoked by repetitive nerve stimulation (RNS) of the tibial nerve at a high frequency (50 Hz). The decrement-increment phenomenon is indicated by the arrow.

Neurophysiologic studies performed in the proband and in his affected mother showed the presence of repetitive components of CMAP in ulnar as well as in tibial nerves. They were evident both on routine motor studies and on F-wave studies, making F-waves unrecognizable. Sensory conduction studies were unremarkable. In order to investigate the neuromuscular transmission (NMT) in these patients, we also performed repetitive nerve stimulation tests at 3, 20 and 50 Hz, prolonged for 10 stimuli. Repetitive nerve stimulation tests at 3 Hz did not result in any significant variation in CMAP amplitude. Conversely, a train of stimuli at 20 Hz or 50 Hz showed a decrement of the amplitude of the first CMAP elicited followed by an increment of the second CMAP amplitude elicited (decrement-increment phenomenon), similar to what is usually observed in organophosphate intoxication (Fig. 2). These findings confirmed the abnormal neuromuscular transmission exhibited in Kv1.1 knock-in ataxic mice^18^ pointing out the role of juxtaparanodal K^+^ channels in dampening the excitability of motor nerve axons during fatigue.

### Identifying the Mutation as c. 907 T > G, p. F303V

Genetic analysis (Fig. 1b) showed that the patient carries a heterozygous T > G substitution in exon 2 of the *KCNA1* gene at nucleotide 907 (NM_000217.2: c.907T > G) resulting in a substitution of phenylalanine at codon 303 by a valine (p.F303V). Protein-homology analysis identified a highly evolutionary conserved phenylalanine at position 303 (Fig. 1d) located within the S4 segment of the voltage-sensor portion of the Kv1.1 channel (Fig. 1c). According to PolyPhen-2 tool, the mutation was probably damaging with a score 0.999. According to SIFT, the mutation was deleterious with a score 0.001.

### F303V reduces whole-cell Kv1.1 current amplitude

**Figure 3:**
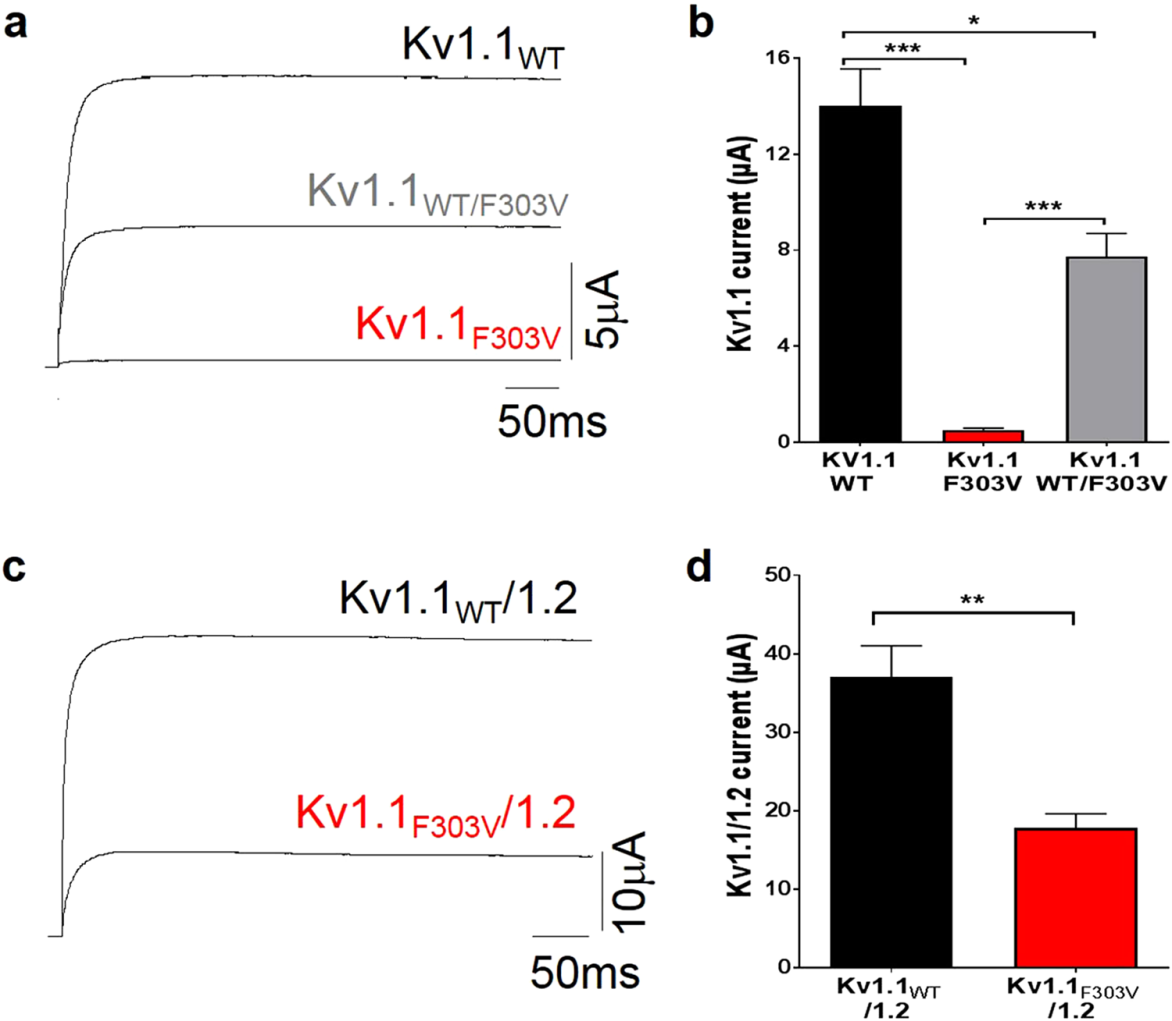
Current reduction in cells expressing F303V cRNA. Representative Kv1.1 (**a**) and Kv1.1/1.2 (**c**) current traces recorded 5 days after oocyte injection at +60 mV from a holding potential of −80 mV. Bar graphs showing mean reduction in (**b**) Kv1.1 (n = 10 cells) and (**d**) Kv1.1/1.2 (n = 8) current amplitudes by the mutation (*p < 0.005, **p < 0.001, ***p < 0.0001).

For current measurements, oocytes were depolarized from a holding potential of −80 mV to a voltage of +60 mV for 500 ms. Currents and mean values reported are from recordings taken 5 days after injection, *i.e*. during peak mutant expression thereby allowing for a better mutant current bioanalysis (see Supplementary section, Figure S1). Cells injected with homomeric Kv1.1_WT_ cRNA (50 nl, 5 ng/µl) showed robust potassium delayed-rectifying currents (14 ± 1.6 µA, n = 10) that were significantly greater than those recorded from cells expressing the same amount of homomeric Kv1.1_F303V_ channels (0.5 ± 0.08 µA, p < 0.0001 n = 10; Fig. 3a and b). In the heterozygous state both normal and mutant alleles may be expressed. Co-injecting equal amounts of Kv1.1_WT_ and Kv_F303V_ cRNA (2.5 ng/µl each, 50 nl total injected volume) in oocytes allows for the co-expression of wild-type and mutant subunits thereby possibly mimicking the heterozygous state of the patient. The mean current obtained when both subunits were co-expressed in a 1:1 ratio was 7.7 ± 1 µA (n = 10), a significantly smaller mean current than that obtained from homomeric WT channels (p < 0.005) and larger than that from homomeric mutant channels (p < 0.0001: Fig. 3a and b). On the other hand, currents from heteromeric Kv1.1/1.2 channels, co-expressing F303V with Kv1.2 in a 1:1 ratio, resulted in a whole-cell current mean (13 ± 1.3 µA, n = 8) at +60 mV that was significantly smaller than that of the control Kv1.1_WT_/1.2 (37 ± 4 μA, n = 8, p < 0.001; Fig. 3c and d).

### Effect of F303V on voltage-dependent activation of Kv1.1 channels

**Figure 4:**
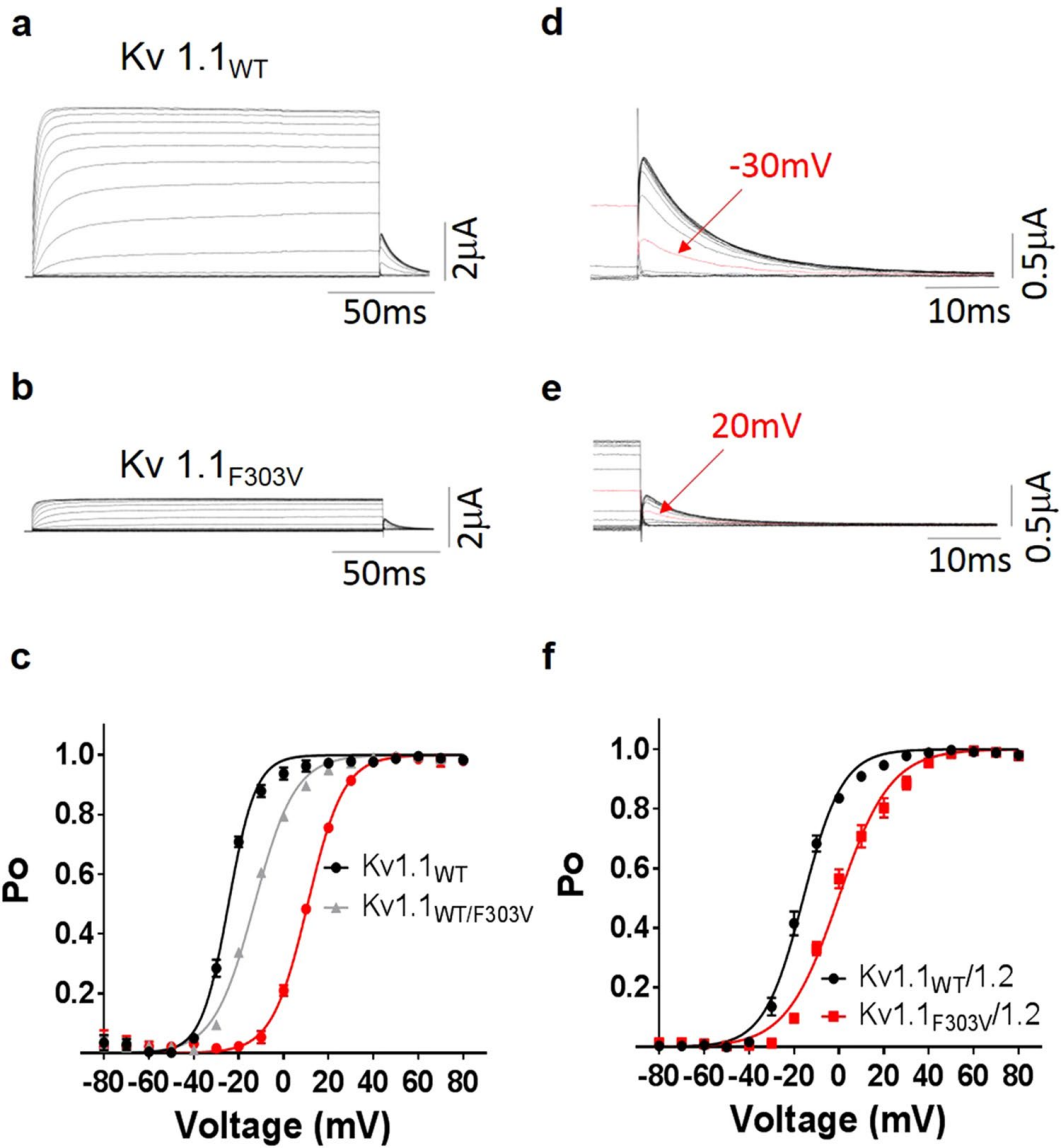
Effect of F303V on the voltage-dependence of current activation. Representative Kv1.1_WT_ (**a**) and Kv1.1_F303V_ (**b**) whole-cell current families. Tail currents for Kv1.1_WT_ (d) and Kv1.1_F303V_ (e) were recorded at −50 mV preceded by several pre-pulse voltage commands. Arrows point out tail current amplitudes recorded after the indicated pre-pulse voltage commands. Kv1.1 (**c**) and Kv1.1/1.2 (**f**) activation (Po-V) curves derived from peak amplitudes of tail currents plotted as a function of pre-pulse potentials, and fitted with a Boltzmann function. For homomeric and heteromeric channels F303V mutation resulted in a right shift of the open probability curve to more positive potentials.

To analyze the effect of the mutation on the voltage-dependence of Kv1.1 current activation, tail current amplitudes were recorded at −50 mV after 200 ms pre-pulse commands from −80 to +80 mV (Fig. 4). The peak amplitudes of the tail currents (Fig. 4d and e) were plotted as a function of the pre-pulse potential and the mean values were fitted with the Boltzmann function Po(V) = 1/{1 + exp[(V_1/2_ − V)/*k*]}, where V_1/2_ is the half-maximal voltage, and *k* the slope factor. The F303V mutation resulted in a significant change in the open probability (*P*o–V) curve and hence in a 36 mV right shift of the V_1/2_ value to more positive potentials (Fig. 4c and e). Kv1.1_WT/F303V_ heteromers had a *P*o–V curve intermediate in position between the Kv1.1_WT_ and Kv_F303V_ homomer curves, and had a significantly different V_1/2_ compared to the wild type and the homomer (Fig. 4c; Table 1). The slope factor, derived from the Boltzmann fit of tail currents, was greater for the Kv_F303V_ and Kv1.1_WT/F303V_ curves than that of the Kv1.1_WT_ (Table 1). A mutation-induced shift to more positive potentials (16 mV shift) and increases in slope factor were also obtained for the Kv1.1_WT_/Kv1.2 heteromer (Fig. 4f; Table 1).

**Table 1:** Biophysical parameters.

Channel	Activation		Inactivation		Window		Recovery	
	V_1/2_ (mV)	*k*	V_1/2_ (mV)	*k*	Peak (mV)	Area	*τF* (ms)	*τS* (s)
Kv1.1_WT_	−25 ± 0.3	6 ± 0.3	−38 ± 0.3	3 ± 0.3	−33	1	64 ± 14	2.5 ± 0.2
Kv1.1_WT/F303V_	−13 ± 0.3*	9 ± 0.3*	−34 ± 0.4*	4 ± 0.3	−27	0.8	69 ± 18	2.6 ± 0.3
Kv_F303V_	+11 ± 0.4*	8 ± 0.4*	−18 ± 0.5*	5 ± 0.5^†^	−7	0.4	30 ± 6*	2.6 ± 0.2
Kv1.1_WT_/1.2	−16 ± 0.4	9 ± 0.3	−31 ± 0.9	4 ± 0.9	−26	1	110 ± 2	6 ± 0.1
Kv_F303V_/1.2	0 ± 0.5^#^	12 ± 0.4^#^	−22 ± 0.6^#^	5 ± 0.5	−16	0.6	44 ± 5^#^	2 ± 0.1^#^

*V*_1/2_ and *k* represent voltage for half-maximal activation and slope factor determined from Boltzmann fits; mean ± SE. n = 6 for each channel group. ^*,†^Significantly different from Kv1.1WT (*p < 0.0001, ^†^p < 0.005). ^#^Significantly different from Kv1.1_WT_/1.2 (p < 0.0001).

### Effect of F303V on activation and deactivation kinetics

**Figure 5:**
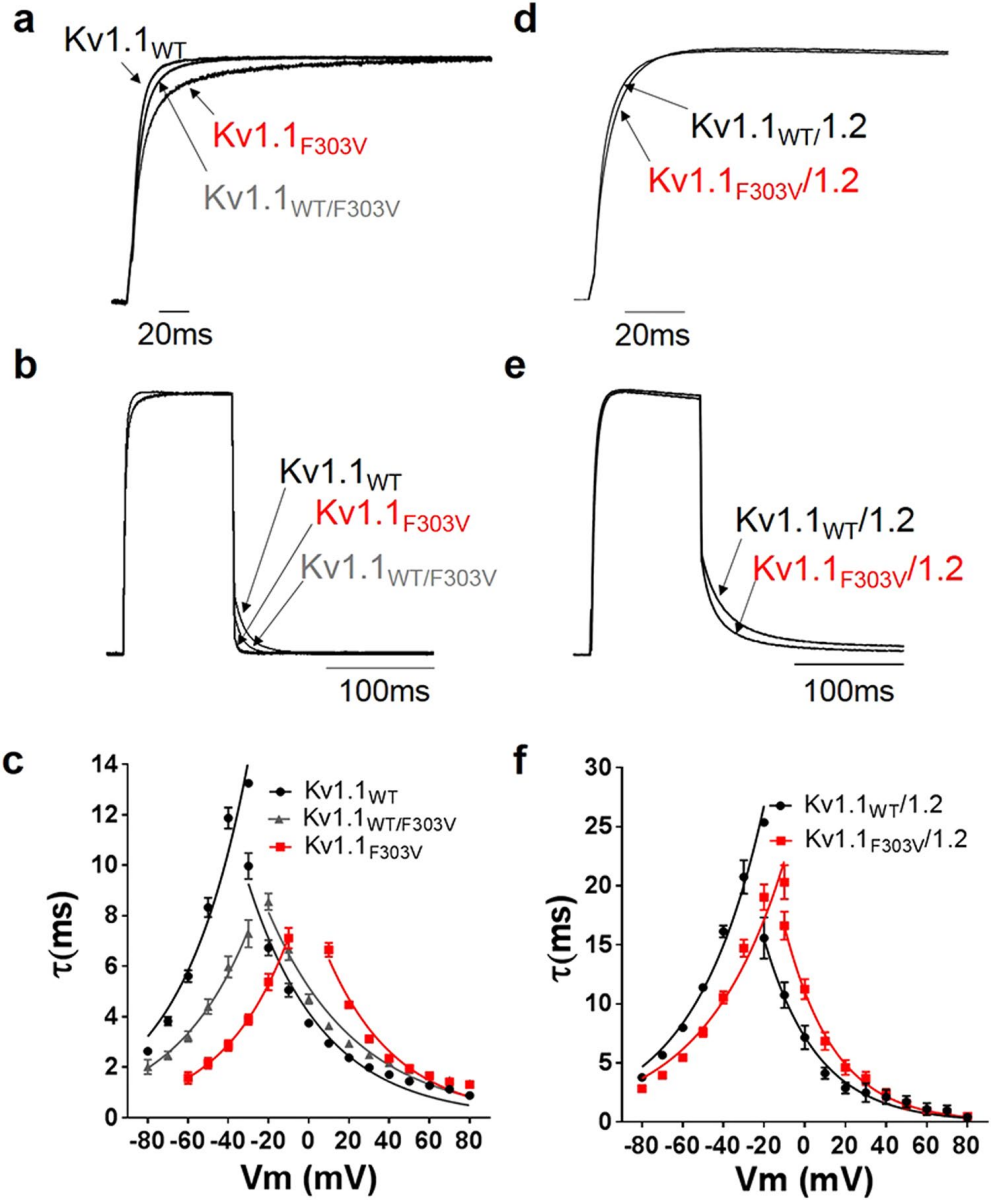
Effect of mutation on channel activation and deactivation kinetics. Activation kinetics were derived from the rising phase of currents evoked by depolarizing pulses (−20 to +80 mV), lasting 300 ms from a holding potential of −80 mV. Deactivation kinetics were derived from tail currents recorded at potentials −20 to −80 mV (pre-pulse potential +20 mV). Normalized and superimposed representative Kv1.1 (**a**) and Kv1.1/1.2 (**d**) activating currents were recorded at +20 mV, and Kv1.1 (**b**) and Kv1.1/1.2 (**e**) deactivating currents recorded at −50 mV. Activating and deactivating current traces were fitted with double and single exponential functions, respectively. Time constants of the currents were plotted as a function of membrane potential for Kv1.1 (**c**) and Kv1.1/1.2 (**f**) channels. Plots show that mutation results in slower activation but faster deactivation. The data points are mean ± SE of 8 cells.

To determine changes in activation and deactivation kinetics, activation time constants derived from double exponential fits of the rising phase of the test currents and deactivation time constants from single exponential fits of tail currents were plotted as a function of membrane potential (Fig. 5c and f). The plotted data points were fitted with the equation τ = τ_V1/2_ exp{(V-V_1/2_)/k}, where τV_1/2_ is the time constant at the V_1/2_ of the channels and k is the slope factor for the voltage dependence of the time constants. Kinetic analysis revealed that, compared to Kv1.1_WT_ currents, a slower activation yet faster deactivation was observed for Kv1.1_F303V_ and Kv1.1_WT/F303V_ currents (Fig. 5a-c). The same change in gating kinetics, albeit smaller, was observed for the F303V mutation in Kv1.1/1.2 heterotetramer (Fig. 5d-f).

### Effect of mutation on the voltage-dependence of slow inactivation and window current

**Figure 6:**
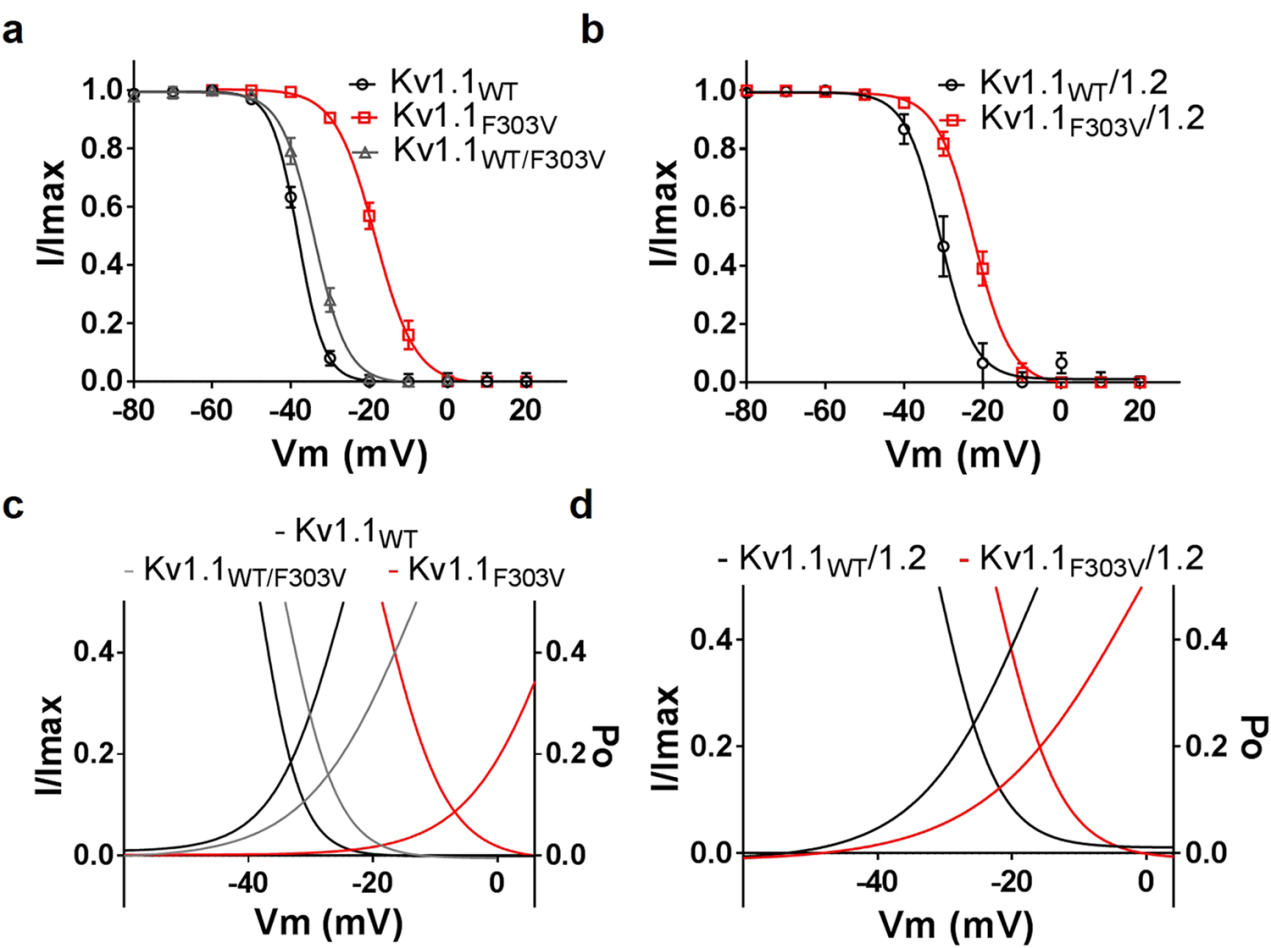
Effect of mutation on voltage-dependence of slow inactivation of Kv1.1 channels and on window currents. Cells were depolarized to various pre-pulse potentials, from −80 mV to +20 mV in +10 mV increments for 20 seconds, and then held at +40 mV test potential for 200 msec. Peak current amplitudes recorded at test potential of +40 mV were normalized (I/Imax) and these values were plotted as a function of the pre-pulse potentials and fitted with a Boltzmann function. The mutation resulted in the positive shift of the inactivation-voltage relationships for both homomeric (**a**) and heteromeric (**b**) channels. F303V reduced both (**c**) Kv1.1 and (**d**) Kv1.1/1.2 window currents (the triangular areas under the overlapping point of the activation and inactivation curves of the respective channel).

As in the voltage-dependence of activation, the mutation right shifted the inactivation curves (Fig. 6a and b) towards more positive values. The V_1/2_ of the slow inactivation for Kv1.1_F303V_ was right shifted 20 mV compared to Kv1.1_WT_ (Table 1). As for the inactivation of the Kv1.1/1.2 heterotetramers, the V_1/2_ was 9 mV more positive. On the other hand, the slope factor of inactivation curve was statistically different from the wild-type only for the homotetrameric Kv1.1_F303V_ curve (Table 1).

The window current, a measure of the number of inactivating ion channels that are tonically active, was quantified by using an integration gadget (OriginLab, Northampton, MA) to integrate the overlapping triangular area between steady-state activation and inactivation curves (Fig. 6c and d). This overlapping area was smaller in the curves derived from oocytes expressing the mutation, both in the Kv1.1_F303V_ homotetrameric (2/5^th^ the area of the WT) and Kv1.1_WT/F303V_ heterotetrameric (4/5^th^ that of the WT) channels (Fig. 6c; Table 1). This reduction in area was also apparent in curves derived from Kv1.1_F303V_/Kv1.2 when compared to its WT control counterpart (Fig. 6d; Table 1). The voltage point whereby the Kv1.1_WT_ activation and inactivation curves intersect (the peak of the triangle) were right shifted by the mutation to more positive potentials. Specifically, there was a 26 mV shift for Kv1.1_F303V_ and a 6 mV shift for the co-expressed Kv1.1_WT_/Kv1.1_F303V_. As for the Kv1.1_F303V_/Kv1.2 heterotetramers, there was a similar 10 mV positive shift away from its wild-type Kv1.1/Kv1.2 control counterpart.

### Effect of mutation on slow inactivation and recovery

**Figure 7:**
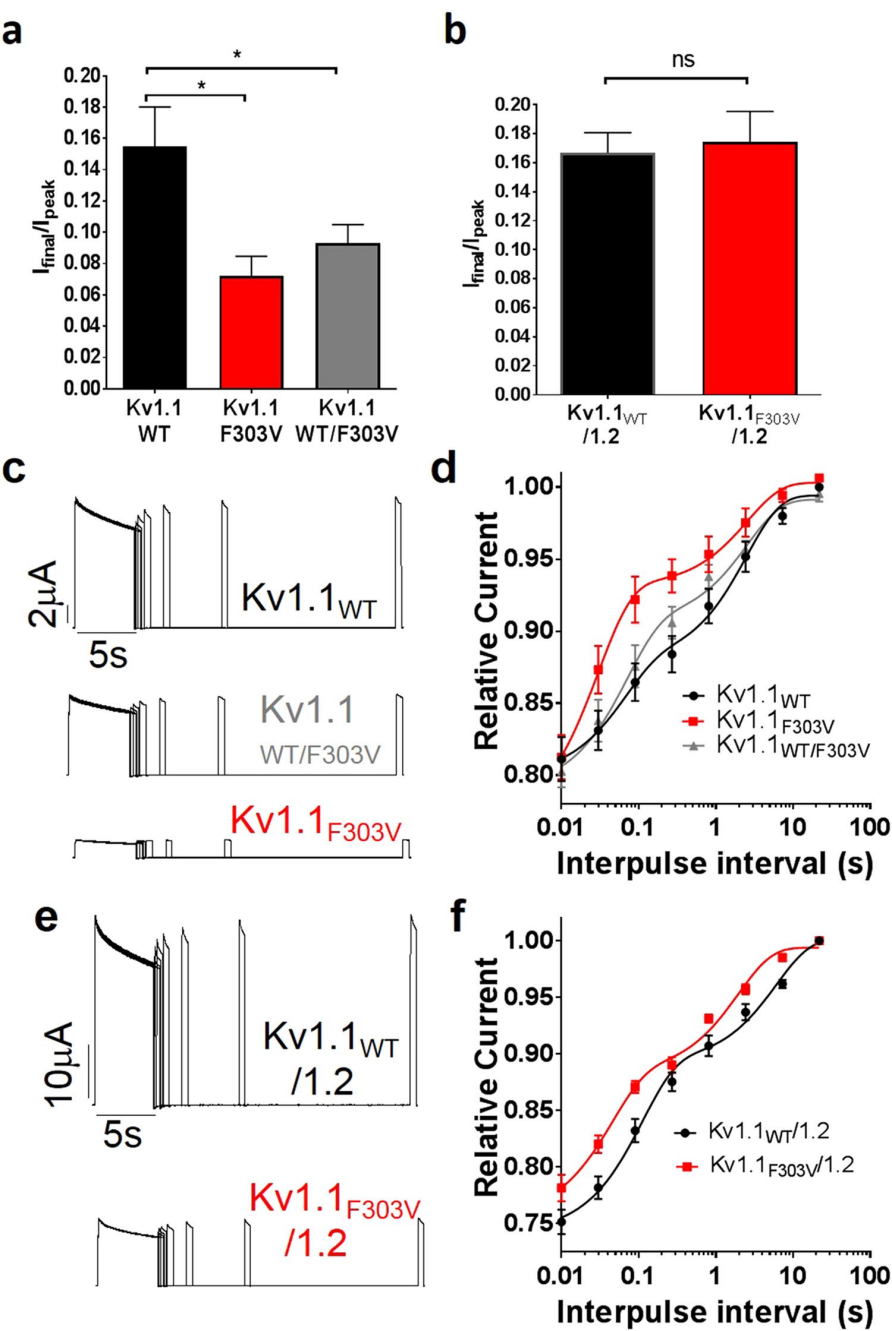
Effect of F303V mutation on slow inactivation, residual current and recovery. (**a** and **b**) Bar graphs showing residual current after slow inactivation for Kv1.1 (**a**) and Kv1.1/1.2 (**b**) channels. A double-pulse recovery protocol at a depolarizing voltage of +60 mV was used to derive currents from (**c**) Kv1.1_WT_ (top), Kv1.1_WT/F303V_ (middle) and Kv1.1_F303V_ (bottom) channels, and from (**e**) Kv1.1_WT_/1.2 (top) and Kv1.1_F303V_/1.2 (bottom) channels. Normalized peak current amplitudes were plotted as a function of interpulse interval for Kv1.1 (**d**) and Kv1.1/1.2 channels (**f**). The solid lines show the fit with the double exponential function Y = Y0 + SpanFast*(1-exp(-KFast*X)) + SpanSlow*(1-exp(-KSlow*X)), where SpanFast = (Plateau-Y0)*PercentFast*.01, SpanSlow = (Plateau-Y0)*(100-PercentFast)*.01 and K is the rate constant. Data points are mean ± SE of 6 cells.

To determine the changes in slow inactivation, the oocytes were depolarized at +60 mV for 3.5 minutes. The decaying phase of the current was then fitted with a double exponential to compute fast and slow kinetics of inactivation. Although there was no apparent change in the slow inactivation (Figure S2), the residual current (final current divided by peak current, I_Final_/I_Peak_), for the Kv1.1 homotetramer was changed by the mutation. The Kv1.1_WT_ channel exhibited more residual current in comparison to the other two groups (p < 0.05, n = 6) (Fig. 7a). We did not observe any difference in the residual current between the Kv1.1_WT_/Kv1.2 and Kv1.1_F303V_/Kv1.2 channels (Fig. 7b). Recovery from inactivation which is an indicator of refractory period, was investigated using a double-pulse protocol whereby consecutive +60 mV pulses lasting 5 seconds are separated by interpulse intervals of increasing durations ranging from 0.010 to 21 s. The peak current amplitudes of the second pulses were divided by the first ones, and the values were then plotted as a function of the interpulse interval and fitted with a double exponential function (Fig. 7d and f). As indicated in the figures, the rate of recovery from inactivation was increased for the channels with the F303V mutation. The τfast time constant of inactivation recovery derived from the channels with the mutation were at least two fold smaller than that of their respective WT controls, indicating a faster recovery. There was no difference in τfast between Kv1.1_WT/F303V_ and Kv1.1_WT_. On the other hand, for τslow, a significant difference was observed only between Kv1.1_F303V_/Kv1.2 and Kv1.1_WT_/Kv1.2 (Table 1).

### *In-silico* Structural analysis

**Figure 8:**
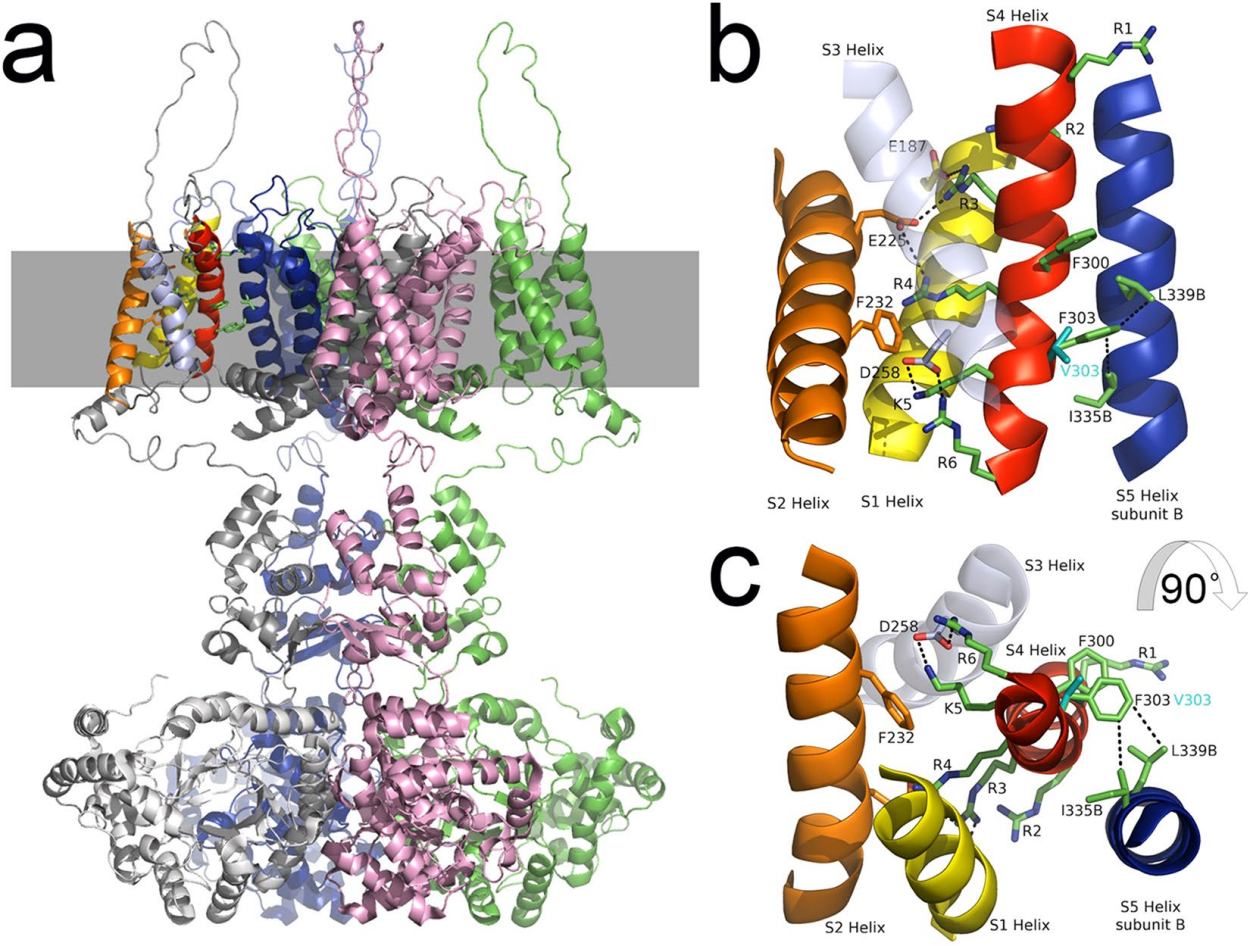
Model structure of the human Kv1.1 potassium channel in the open-state. (**a**) The structure of the tetrameric channel is shown with each of the four polypeptide subunits colored overall grey, green, pink and blue. The position of the membrane is illustrated by a grey band and the brightly colored helices in the top left of the figure are shown in the expanded Figures b and c. (**b**) The membrane spanning region of interest depicting four helices of one subunit (S1 yellow, S2 orange, S3 lavender and S4 red) and one helix from a neighboring subunit (S5B, blue) are shown. Helix S3 lies in front of the other helices and is rendered transparent for clarity. Significant side chains are drawn in stick representations and binding partners indicated by black dotted lines between them. These include R3-E187, R3-E225, R4-E225, K5-D258, R6-D258, F303-L339B and F303-I335B. The predicted position of the V303 mutant side chain is also shown. (**c**) Illustrates the same structure as depicted in B rotated ninety degrees towards the observer. The stacking of F300 and F303 is well illustrated in this figure.

The F303V mutation reported here occurs on the important and mobile helix S4 (Fig. 8, red helix) of the voltage sensing domain and corresponds to the F305 residue in the Kv1.2 crystal structure^39^ (Figure S3). In the open-state, the S4 helix is stabilized by salt bridges formed by voltage-sensing positively charged residues R2, R3, R4, K5 and R6 (dotted lines in Fig. 8b and c, R2 salt bridge is not shown). Our structural analysis shows that the F303 residue is appropriately positioned to interact with L339 and I335 of the S5 helix of a neighboring subunit (indicated as L339B and I335B on S5 helix subunit B in Fig. 8b and c). In addition, both L339 and I335 residues are somewhat exposed to the hydrophobic environment of the lipid bilayer.

## Discussion

In this study, we present a novel mutation in the *KCNA1* gene found in a patient displaying typical signs and symptoms of EA1. The mutation results in the substitution of a phenylalanine with a valine in position 303. The phenylalanine at this position is highly conserved through evolution, thereby emphasising the mutational substitution as a causal role for the symptoms observed and highlighting its importance for normal channel gating.

Consistent with previous reports on genetic defects in *KCNA1* associated with EA1^24^, the Kv1.1_F303V_ mutation resulted in a remarkable *loss of function*, with about 96% macroscopic current decrease when the mutation was expressed alone. The reduction in current amplitude was observed over several days after cRNA injection and at +60 mV, *i.e*. at maximal activation of both WT and F303V channels. This suggests mutant channels may not be as readily expressed in the membrane as the WT channels (possibly due to retention in the endoplasmic reticulum, defective membrane trafficking, or enhanced protein degradation). When F303V was co-expressed with WT Kv1.1 subunits at a 1:1 ratio there was a 55% decrease in current amplitude, and the currents displayed kinetics intermediate between those of homomeric F303V and homomeric WT channels, yet closer to the WT parameters. This is probably because, in co-expression experiments, only a few mutant channels and some heteromeric channels are expected to reach the membrane, making the voltage-dependent parameters closer to those of the WT.

The F303V mutation alters the voltage-dependence and of Kv1.1, shifting the half-maxiamal activation voltage 36 mV towards more positive potentials compared to WT. Although the mutation is positioned in the voltage-sensing S4 segment of the channel, it is still quite surprising that phenylalanine, a neutral non-polar residue, causes such a remarkable impact on gating. While expectedly, a mutation that replaces the positively charged Arg (R307) with a Cys in the S4 segment, reported for another EA1 patient, altered the voltage-depedence of Kv1.1^31^. The F303V-induced depolarizing shift was evident in both the activation and inactivation phases of the channel, and was hence apparent in the window current. Moreover, the F303V mutation resulted in a deceleration of activation kinetics and acceleration of deactivation kinetics confirming a resilient effect on channel gating. These effects were also evident when the F303V mutation was expressed in the Kv1.1/1.2 channel, which is in agreement with a previous study demonstrating altered kinetics and voltage-dependence of Kv1.1/1.2 channels bearing EA1 mutations, thereby confirming the finding that mutations in a single gene coding for a subunit may disrupt the functions of other closely associated subunits^21^.

The recovery from slow inactivation was faster for channels carrying the mutation indicating a destabilization of the inactivated-state. A more rapid recovery means F303V channels will be available faster and contribute more readily towards the generation of new action potentials. Decreased current amplitudes, reduced window currents, increased recovery rate, and depolarizing shifts in the voltage-dependence of Kv1.1 will, altogether, more than likely result in an increase in neuronal excitability that in EA1 would manifest as ataxic attacks and myokymia, as observed in the proband and family members carrying the mutation. As shown previously^21^, an altered delayed-rectifier function as a consequence of EA1 mutation in heteromeric channels comprising Kv1.1 and Kv1.2 subunits, known to be expressed at the presynaptic terminals of basket cells, increases membrane excitability, prolongs action potential duration, and enhances Ca^2+^ ion influx^21, 38^. Larger amounts of γ-aminobutyric acid (GABA) released from basket cell terminals would then alter inhibitory outputs of relevant Purkinje cells likely resulting in ataxia.

Of particular interest is the presence of nystagmus in the extreme lateral gaze of the proband’s family member pt II1. Although nystagmus has not been clearly associated thus far with EA1, its appearance as a symptom here is not completely surprising. After all, vestibulogenic stimulations trigger attacks of ataxia in EA1 patients and the vestibular system is an established regulator of our sense of balance, position in space, adaptive control of posture and a number of eye movements including the vestibulo−ocular reflexes. There is evidence for the abundant expression of Kv1.1 channels in both vestibular nuclei^45^ and ganglion cells^46^ where they, along with other distinct K^+^ channels, control the excitability, discharge pattern and resonance properties of neurons of the vestibular system^47, 48^. Accordingly, the nystagmus may be the result of a mutation-induced effect on vestibular function.

Altered Ca^2+^ homeostasis was observed in the motor axons of Kv1.1_V408A/+_ mice, a rodent model of EA1^18^. By investigating the neuromuscular transmission of these ataxic mice and their susceptibility to physiologically relevant stressors, direct evidence was provided for the generation of neuromyotonic/myokymic activity by motor nerve axons with dysfunctional Kv1.1/Kv1.2 channels and altered Ca^2+^ signals. Furthermore, this study also pointed out the significant role that juxtaparanodal K^+^ channels composed of Kv1.1/Kv1.2 subunits play in dampening the excitability of motor nerve axons during fatigue or ischemic insults^18^. Collectively, these findings are consistent with the abnormal neuromuscular transmission exhibited by our proband during resting conditions (myokymia), physical exercise or when challenged with high frequency stimuli to ulnar and tibial nerves.

Channel kinetics can be assessed with a voltage protocol in which the membrane is depolarized to open the channels and then hyperpolarized to close them. In general, the rate of closure is voltage dependent; it becomes faster as the membrane voltage is made more negative. The assessment of the time constants of deactivation as a function of voltage for wild-type channels clearly shows (Fig. 5) that the more negative the repolarization potential the faster the closure of the channels. However, when F303 is replaced by a valine the channels take a much shorter time to close. The negative Vm and faster closure suggest that the Phe to Val substitution energetically favors a closed channel state relative to an open one. This emphasizes the importance of a Phe side chain at this position for proper conformational equilibria of the channel. In *Shaker* channels, the presence of an intrinsically repulsive electrostatic interaction between the face of the aromatic residue F481 and the net negative charge of the acidic side chain E395 (located on an adjacent subunit) that may serve to inherently destabilize the channel open state has been previously proposed^37^. Moreover, we have previously shown that the EA1 mutation E325D (corresponding to E395 in *Shaker* channels) alters the open-state stability of both homomeric Kv1.1^23^ and heteromeric Kv1.1/Kv1.2^21^ channel types. Likely, these effects could account for an altered interaction between the E325 residue with either F411 (Kv1.1) or F413 (Kv1.2) in the adjacent S6 segment (corresponding to F481 in *Shaker* channels) in accordance with the findings discussed above^37^. Thus, open-state destabilization via aromatic-dependent interactions appears a mechanism through which EA1 mutations alter channel function and result in disturbed electrical excitability and prolonged action potential repolarization in excitable cells^21, 38^.

The mutation reported here occurs on the mobile helix S4 that is stabilized by salt bridges formed by the voltage-sensing positively charged residues R2, R3, R4, K5 and R6 when the channel is in the open position (see Fig. 8). We show here that the structure is further stabilized by hydrophobic interactions between side chains including F300 and F303 which lie in a staggered arrangement in the S4 3_10_ helix (Fig. 8c). Such arrangements of aromatic amino acids near the C terminal ends of alpha helices has been shown to have a stabilizing influence^44^. In particular, the structural analysis indicates that F303 interacts with L339 (F303 CG1 to L339 CD1 is 4.2 Å) and I335 (F303 CE1 to I335 CG2 is 4.5 Å) residues positioned in the S5 helix of a neighboring subunit and, since these latter residues are somewhat exposed to the hydrophobic environment of the membrane, they probably also contribute stabilizing interactions with the phospholipids therein. The substitution of F303 by the smaller valine in the F303V mutant will either reduce or abolish the effectiveness of any of these interactions (V303 CG1 to L339 CD1 is 6.8 Å, V303 CG2 to I335 CG2 is 6.7 Å), resulting in a less stable open- and inactivated-state of the channel. The remarkable effect of the mutation also on the voltage-dependence and activation kinetics of the channel suggests that the abnormal interaction of V303 with L339 and I335 will also perturb the transient salt bridges formed by the gating-charge residues with acidic residues on S1 and S2 (Fig. 8) and with lipid negatively charged phosphodiester groups during activation, impeding the transitions underlying opening and reducing the voltage sensitivity of the channel.

During the closing of the channel, helix S4 is considered to undergo large conformational changes including rotation (up to 120°; anticlockwise as viewed in Fig. 8c) and translation (approximately 10 Å; downwards as viewed in Fig. 8b)^49^. This movement has to occur within the confines of the membrane, within the VSD core formed by helices S1, S2 and S3 and is powered by the hyperpolarization of the membrane. During this process arginine R1 becomes proximal to phenylalanine F232. Moreover, it is also assumed that the 3_10_ helix reverts to a more favorable alpha helical conformation during this process which would misalign the stacked arrangement of F300 with F303. It is tempting to speculate that the bulky side chain of phenylalanines may restrict this motion and that replacement with valine at position 303 could expedite the open to the closed transitions. Whether or not valine at position 303 may stabilize the closed resting state of the channel remains undeterminable due to the lack of crystal structures of the channel in the closed conformation.

In conclusion, we found a new mutation in *KCNA1* (F303V) and demonstrated that the reduced current amplitudes and altered gating properties of the channel account for its pathophysiological impact. Remarkably, our findings indicate that the presence of an aromatic residue (phenylalanine) adjacent the positively charged residue (Lys 304) in the S4 segment, is crucial for proper Kv1.1 channels gating likely by controlling the overall interactions taking place between S4 and the neighboring alpha helixes. This may be regarded as a new mechanism by which an aromatic residue fine-tunes conformational equilibria in Kv1.1 channels. In a much broader sense, understanding these “*experiments*” of Nature that result in overt channelopathies is helping us to unravel the molecular workings of the channels involved.

## Electronic supplementary material

Supplementary Information
