# Supplementary material for: A channelopathy mutation in the voltage-sensor discloses contributions of a conserved phenylalanine to gating properties of Kv1.1 channels and ataxia
Source: Sci Rep. 2017 Jul 4;7:4583. doi: 10.1038/s41598-017-03041-z (PMC5496848; doi:10.1038/s41598-017-03041-z)
Supplement: Supplementary file 1 — Supplementary Information [file 41598_2017_3041_MOESM1_ESM.doc]

**A channelopathy mutation in the voltage-sensor discloses contributions of a conserved phenylalanine to gating properties of Kv1.1 channels and ataxia**

Sonia Hasan^1,+^, Cecilia Bove^2,+^, Gabriella Silvestri^3^, Elide Mantuano^4^, Anna Modoni^3^, Liana Veneziano^4^, Lara Macchioni ^2^, Therese Hunter^5^, Gary Hunter^5^, Mauro Pessia^2,5^, Maria Cristina D’Adamo^5,*^

Figure S1: Time course of Kv1.1 expression. Oocytes were depolarized from a holding potential of -80mV to a voltage of +60mV for 500ms. Current amplitudes were plotted as a function of days after oocytes were injected. The data points are mean ± SE of 10 cells.


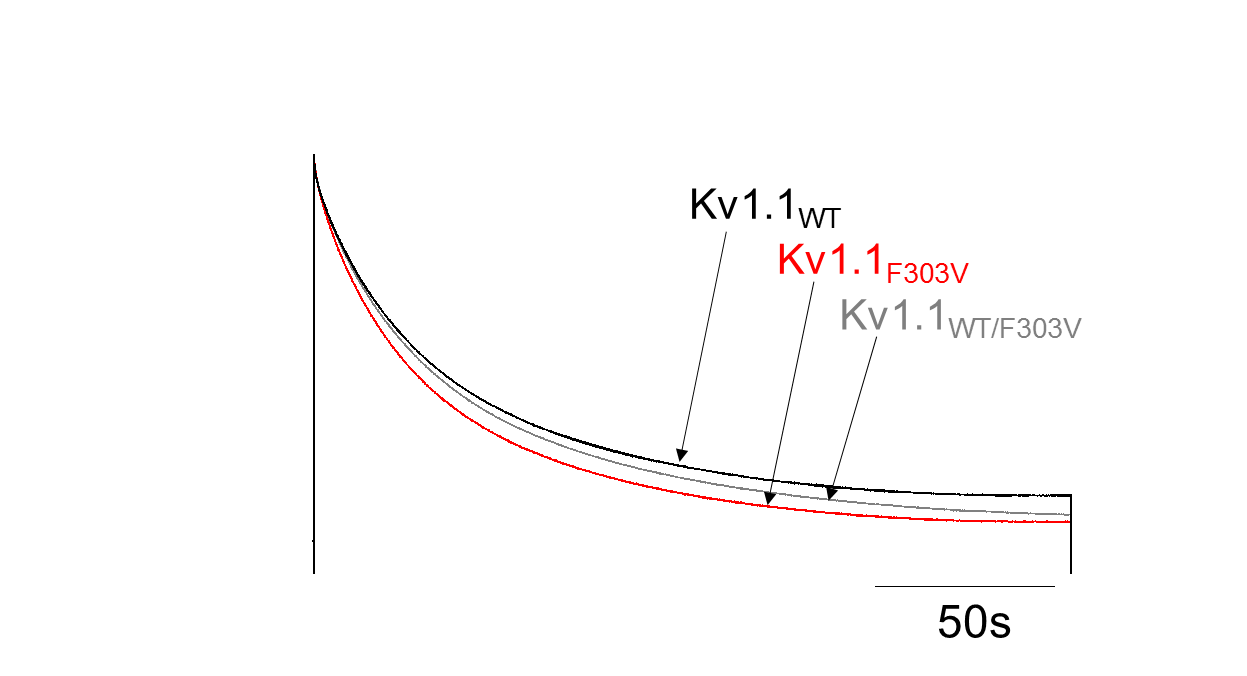


Figure S2: Effect of mutation on slow inactivation. Normalized and overlaid current traces showing the slow inactivation time course for the indicated channels. The membrane potential of oocytes were held at -80 mV and current traces were evoked by depolarizations at +60 mV for 3.5min. I_peak_ is the instantaneous current whereas I_final_ is the current amplitude measured at the end of the depolarizing step. The traces were fitted with two exponential functions and no statistical differences were found in either τ_fast_ or τ_slow_ of all channel types (data not shown).

Kv1.1 1 mtvmsgenvdeasaapghpqdgsyprqadhddheccervvinisglrfet 50

Kv1.2 1 mtvatgdpvdeaaalpghpqd-tydpea---dheccervvinisglrfet 46

Kv1.1 51 qlktlaqfpntllgnpkkrmryfdplrneyffdrnrpsfdailyyyqsgg 100

Kv1.2 47 qlktlaqfpetllgdpkkrmryfdplrneyffdrnrpsfdailyyyqsgg 96

Kv1.1 101 rlrrpvnvpldmfseeikfyelgeeamekfredegfikeeerplpekeyq 150

Kv1.2 97 rlrrpvnvpldifseeirfyelgeeamemfredegyikeeerplpenefq 146

Kv1.1 151 rqvwllfeypessgparviaivsvmvilisivifcletlpelkdd-kdft 199

Kv1.2 147 rqvwllfeypessgpariiaivsvmvilisivsfcletlpifrdenedmh 196

Kv1.1 200 g---tvhridnttviy-nsniftdpffivetlciiwfsfelvvrffacps 245

Kv1.2 197 gggvtfhtysnstigyqqstsftdpffivetlciiwfsfeflvrffacps 246

Kv1.1 246 ktdffknimnfidivaiipyfitlgteiaeq-egnqkgeqatslailrvi 294

Kv1.2 247 kagfftnimniidivaiipyfitlgtelaekpedaqqgqqamslailrvi 296

Kv1.1 295 rlvrvfrifklsrhskglqilgqtlkasmrelgllifflfigvilfssav 344

Kv1.2 297 rlvrvfrifklsrhskglqilgqtlkasmrelgllifflfigvilfssav 346

Kv1.1 345 yfaeaeeaeshfssipdafwwavvsmttvgygdmypvtiggkivgslcai 394

Kv1.2 347 yfaeaderdsqfpsipdafwwavvsmttvgygdmvpttiggkivgslcai 396

Kv1.1 395 agvltialpvpvivsnfnyfyhretegeeqaqllhvss-pnlasdsdlsr 443

Kv1.2 397 agvltialpvpvivsnfnyfyhretegeeqaqylqvtscpkipsspdlkk 446

Kv1.1 444 -rssstmskseymeieedmnnsiahyrqvnirtancttanqncvnkskll 492

Kv1.2 447 srsastisksdymeiqegvnnsnedfreenlktanctlantnyvnitkml 496

Kv1.1 493 tdv 495

Kv1.2 497 tdv 499

Figure S3: Human Kv1.1 protein sequence aligned with rat Kv1.2. Alignment was performed using EMBOSS^1^ Needle alignment program. Kv1.1 F303 and its corresponding Kv1.2 are highlighted in yellow. The L339 and I335 residues and the corresponding Kv1.2 L341 and I337 are highlighted in blue.

**Reference**

1. Rice, P., Longden, I. & Bleasby, A. EMBOSS: The European Molecular Biology Open Software Suite. *Trends Genet* **16**, 276-277 (2000).
